# Age- and Sex-Specific Changes in CMR Feature Tracking-Based Right Atrial and Ventricular Functional Parameters in Healthy Asians

**DOI:** 10.3389/fcvm.2021.664431

**Published:** 2021-06-04

**Authors:** Shuang Leng, Jiajun Guo, Ru-San Tan, Ping Chai, Lynette Teo, Marielle V. Fortier, Chao Gong, Xiaodan Zhao, Ching Ching Ong, John C. Allen, Wen Ruan, Angela S. Koh, Teng Hong Tan, James W. Yip, Ju Le Tan, Yucheng Chen, Liang Zhong

**Affiliations:** ^1^National Heart Centre Singapore, Singapore, Singapore; ^2^Cardiology Division, Department of Medicine, West China Hospital, Sichuan University, Chengdu, China; ^3^Duke-NUS Medical School, Singapore, Singapore; ^4^Department of Cardiology, National University Heart Centre, Singapore, Singapore; ^5^Yong Loo Lin School of Medicine, National University of Singapore, Singapore, Singapore; ^6^Department of Diagnostic Imaging, National University Hospital, Singapore, Singapore; ^7^KK Women's and Children's Hospital, Singapore, Singapore; ^8^Singapore Institute for Clinical Sciences, A*STAR, Singapore, Singapore

**Keywords:** cardiovascular magnetic resonance, feature tracking, right ventricular function, right atrial function, age, sex

## Abstract

Cardiovascular magnetic resonance (CMR) is the reference standard for non-invasive assessment of right-sided heart function. Recent advances in CMR post-processing facilitate quantification of tricuspid annular (TA) dynamics and longitudinal strains of the right ventricle (RV) and right atrium (RA). We aimed to determine age- and sex-specific changes in CMR-derived TA dynamics, and RV and RA functional parameters in healthy Asian adults. We studied 360 healthy subjects aged 21–79 years, with 30 men and 30 women in each of the six age groups. Functional parameters of RV and RA were measured on standard four-chamber cine CMR using fast feature tracking: (1) TA peak velocities (systolic velocity S′, early diastolic velocity E′, late diastolic velocity A′) and TA plane systolic excursion (TAPSE); (2) RV global longitudinal strain (GLS) and strain rates; and (3) RA phasic longitudinal strains and strain rates. S′ and TAPSE exhibited negative correlations with age. RV GLS was significantly higher in females than in males but not associated with age in both sexes. Females had similar E′, lower A′, and higher E′/A′ ratios compared to males. Positive associations of E′ and E′/A′, and negative association of A′ with age were observed in both sexes. Females had higher RA reservoir and conduit strains compared to males. There were significantly negative and positive associations between RA conduit and booster strains, respectively, with age. Age- and sex-specific reference ranges were established, and associations revealed, for fast CMR feature tracking parameters of right heart function in a large normal Asian population.

## Introduction

The right side of the heart has traditionally received less attention than the left, yet there is a growing body of evidence showing that right heart size and function are perhaps of equal importance in providing diagnostic and prognostic information in a wide range of cardiovascular diseases (1).

Cardiovascular magnetic resonance (CMR) imaging is the current gold standard for quantitation of right ventricular (RV) geometry and function (2). Guidelines recommend CMR for assessment of global RV functional parameters such as RV volumes, ejection fraction (EF), and cardiac output (3). Recent advances in CMR post-processing have facilitated reliable quantitation of tricuspid annular (TA) dynamics and longitudinal strains of the RV and right atrium (RA) using feature tracking (4–8). CMR-based TA systolic velocity (S′), early diastolic velocity (E′), late diastolic velocity (A′), and TA plane systolic excursion (TAPSE) have been reported to be significantly lower in patients diagnosed with heart failure (4), hypertrophic cardiomyopathy (4), repaired tetralogy of Fallot (rTOF) (4, 5), unrepaired atrial septal defect (5), and severe tricuspid insufficiency (6) than in normal controls. RV and RA longitudinal strains can be quantified either by feature tracking of the whole RV and RA endocardium, respectively (9, 10), or more rapidly by tracking discrete anatomical points on standard cine CMR (7, 8). A prior study demonstrated the prognostic utility of impaired fast CMR feature tracking-derived RA strain parameters for predicting clinical and hemodynamic deterioration in patients with pulmonary arterial hypertension (PAH) (7). The quantitative RV fast strain and strain rate parameters assessed from CMR identify abnormalities of RV function in rTOF and PAH and are predictive of exercise capacity, RV decompensation, and clinical risks in these patients (8).

The emerging importance of these parameters motivates efforts to define normal ranges and distributions in a healthy population. In addition, most CMR normal ranges for right heart size and volume have been established for Caucasians and may not be generalizable to other ethnicities (11). Accordingly, the aim of this study is to determine age- and sex-specific changes in CMR-derived RV and RA dimensions and functional (systolic and diastolic) parameters among healthy Asian subjects.

## Materials and Methods

### Study Population

In this multicenter study, 360 subjects (aged 21–79 years, 180 males and 180 females) without known cardiovascular disease were enrolled from three hospitals between 2014 and 2019. The subjects were recruited from the (1) Cardiac Aging study (12), (2) INITIATE study, and (3) Database of healthy controls in West China Hospital. All subjects (1) had no known cardiovascular disease and demonstrated no signs of cardiovascular disease in prior electrocardiographic or echocardiographic investigations, (2) displayed no uncontrolled cardiovascular risk factors at the time of enrollment (systolic blood pressure ≤140 mmHg and diastolic blood pressure ≤90 mmHg without anti-hypertensive treatment, total cholesterol <6.2 mmol/L, fasting glucose <7 mmol/L, body mass index <30 kg/m^2^, and current non-smoker status), and (3) had no significant kidney or lung disease. Other exclusion criteria were cerebrovascular disease or nervous system disease, cancer, autoimmune diseases, recent systemic infection (within a month), recent surgery or severe trauma (within a month), any recent medications, and a history of implantation of a pacemaker or other metals that are contraindicated for CMR. The institutional review board of each hospital approved the study protocol. Informed consent was obtained from each participant.

### CMR Acquisition

CMR acquisitions were performed using a 3T magnetic resonance scanner (Ingenia, Philips Healthcare, The Netherlands) at National Heart Centre Singapore, a 1.5T MAGNETOM Aera magnetic resonance scanner (Siemens Healthineers, Erlangen, Germany) at National University Hospital Singapore, and a 3T MAGNETOM Tim Trio magnetic resonance scanner (Siemens Healthineers, Erlangen, Germany) at West China Hospital (Sichuan China). End-expiratory breath-hold-balanced steady-state free precession cine images were acquired in standard short- and long-axis views. Typical parameters for the Philips scanner were as follows: repetition time (TR)/echo time (TE), 3/1 ms; matrix, 240 × 240; flip angle, 45°; field of view, 300 × 300 mm; pixel spacing, 1.25 × 1.25 mm; slice thickness, 8 mm; number of frames, 30/40 per cardiac cycle. Parameters for the 1.5T Siemens scanner were: TR/TE, 33/1 ms; matrix, 192 × 180; flip angle, 58°; field of view, 320 × 300 mm; pixel spacing, 1.67 × 1.67 mm; slice thickness, 6 mm; number of frames, 30 per cardiac cycle. Parameters for the 3T Siemens scanner were: TR/TE, 3.4/1.3 ms; matrix, 192 × 162; flip angle, 50°; field of view, 320 × 270 mm; pixel spacing, 1.67 × 1.67 mm; slice thickness, 8 mm; and number of frames, 25 per cardiac cycle.

### Echocardiography

A sub-study was conducted to validate CMR-derived TA dynamics against those measured using echocardiography. Echocardiography was performed on the same day as CMR using a commercial ultrasound system (Aloka α10, Japan). TA velocities and displacement were measured using tissue Doppler imaging (TDI) and M-mode, respectively, in the apical four-chamber view.

### CMR Data Analysis

The parameters measured were (1) RV volumes and RVEF (derived from standard CMR volumetric analysis), (2) Two-dimensional chamber dimensions (RV chamber diameters and RA area and diameters), (3) TA velocities (systolic velocity S′, early diastolic velocity E′, and late diastolic velocity A′) and displacement (TAPSE), (4) RV longitudinal strain and strain rates, and (5) RA phasic (reservoir, conduit, and booster) longitudinal strains and strain rates.

### Volumetric Analysis and Chamber Dimensions

**Figure 1:**
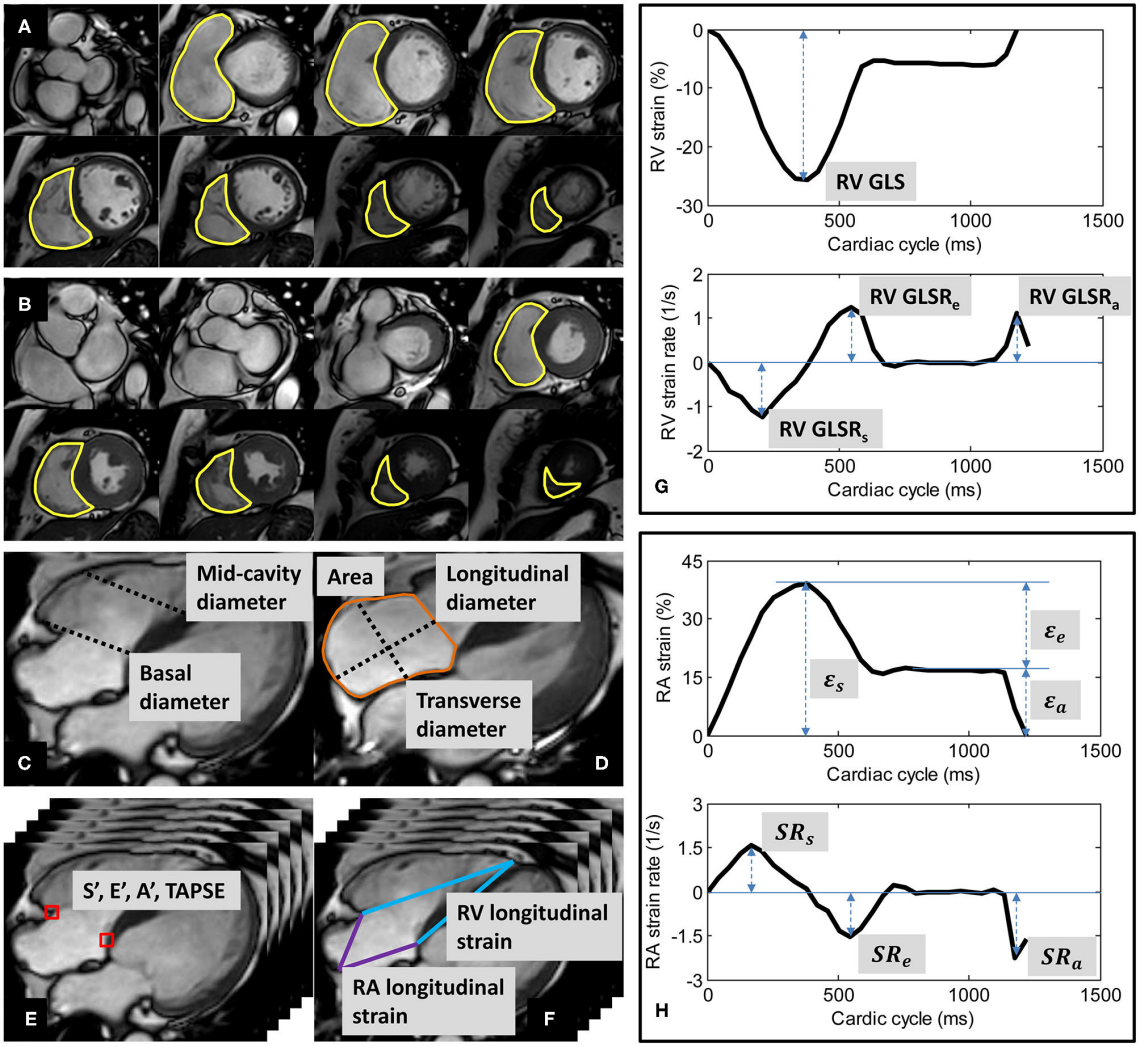
Derivation of right ventricular (RV) volumes by contouring of RV at **(A)** end-diastole and **(B)** end-systole; **(C)** RV basal and mid-cavity diameters at end-diastole; **(D)** right atrial (RA) area, longitudinal and transverse diameters at RV end-systole; **(E)** tricuspid annular dynamics at RV medial (septum) and lateral (free wall); **(F)** longitudinal strains of RV and RA; **(G)** Example of RV strain and strain rate curves; **(H)** Example of RA strain and strain rate curves.

Endocardial contours were manually traced from the stack of short-axis cine images to obtain RV end-diastolic volume (EDV) and end-systolic volume (ESV), from which stroke volume (SV) and EF were derived (Figures 1A,B). Papillary muscles and trabeculae were included in the blood volume (3). RV size was measured from the four-chamber view at end-diastole. RV basal diameter was measured at the level of the tricuspid valve, and mid-cavity diameter was measured in the middle third of the RV at the level of the left ventricular (LV) papillary muscle (13) (Figure 1C).

RA area and diameters were measured from the four-chamber view at RV end-systole. RA longitudinal diameter was measured as the distance between the midpoint of the line joining the medial and lateral (free wall) insertions of the tricuspid valve and the posterior wall (roof) of the RA. RA transverse diameter was measured as the orthogonal line bisecting the longitudinal diameter (14) (Figure 1D).

All chamber dimension measurements were reported as both absolute values and indexed values normalized to body surface area (BSA).

### Tricuspid Annular Velocities and Displacement

Custom software developed in the MATLAB environment (MathWorks Inc., MA, USA) and validated in prior studies (4, 7, 8, 15–19) was used to perform the semi-automatic tracking of medial and lateral tricuspid valve insertions in the four-chamber view (Figure 1E). A video of the TA tracking and resultant TA velocity and displacement curves are shown in Supplementary Materials. Peak TA velocities were read off the velocity curve during systole (S′), early diastole (E′), late diastole (A′); TAPSE—TA displacement at end-systole—was read off the displacement curve (4, 7). We reported mean values for S′, E′, A′, and TAPSE averaged from medial and lateral TA measurements.

### Longitudinal Strains of RV and RA

Using the same custom software, the RV epicardial apex and the RA roof point were tracked in the four-chamber view over the cardiac cycle (Figure 1F). We followed the convention of determining RV apex location by its proximity to the LV apex. The RV apex is close to but separated from the LV apex by the interventricular septal wall, which can be seen on the four-chamber view (13). The RA roof was localized to the intersection of the RA posterior wall and the RA longitudinal diameter.

The distance (*L*) from either the medial or the lateral tricuspid valve insertion to the RV epicardial apex on the four-chamber view was measured at any time point (*t*). RV strain value at time *t* with respect to RV end-diastole (time 0) was calculated using the formula for Lagrangian strain: (*L*(*t*)−*L*(0)) × 100/*L*(0) (8). RV global longitudinal strain (GLS)—strain value at end-systole—was read off the strain curve, and peak global longitudinal strain rates at RV systole (GLSR_s_), early diastole (GLSR_e_), and late diastole (GLSR_a_) were calculated as first time-derivatives of the strain curve at the corresponding cardiac phases (8) (Figure 1G).

Similarly, RA longitudinal strain (ε) was derived from the time-varying distances between either the medial or the lateral tricuspid valve insertion and the RA roof point (7). RA reservoir strain (ε_*s*_), conduit strain (ε_*e*_) and booster strain (ε_*a*_) were read off the generated strain curve at RV end-systole, diastasis, and pre-RA systole, respectively, and the corresponding peak strain rates (*SR*_*s*_, *SR*_*e*_, and *SR*_*a*_) calculated as first time-derivatives of the strain curve at the corresponding cardiac phases (7) (Figure 1H).

A video showing the derivation of RV and RA strain is given in Supplementary Materials. Mean RV and RA strain and strain rate parameters averaged from medial and lateral TA measurements were used for all analyses. RV strain and strain rate parameters were presented as absolute values.

### Conventional Feature Tracking Strain

A sub-study was conducted to validate the fast feature tracking-derived RA and RV strain measurements against those derived from conventional CMR RA and RV endocardial feature tracking using dedicated QStrain software (Version 2.0, Medis BV, Leiden, The Netherlands).

### Statistical Analysis

The distribution normality of continuous variables was assessed using the Shapiro-Wilk test. Data were expressed as mean ± standard deviation (SD), and reported across six pre-specified age groups stratified by sex. Age-specific reference limits were defined as mean ± 1.96·SD. Values obtained in females and males were compared using Student's *t*-test. For either sex, linear regression was used to characterize the relationship of right heart measurements with age. Intra- and inter-observer variability of study parameters was studied in a selected subgroup of 20 cases using Bland-Altman analysis and coefficient of variation. Statistical analyses were performed using SPSS software (Version 17.0, IBM, Chicago, IL, USA). *P* ≤ 0.05 indicated statistical significance.

## Results

### Baseline Characteristics and Summary Results

**Figure 2:**
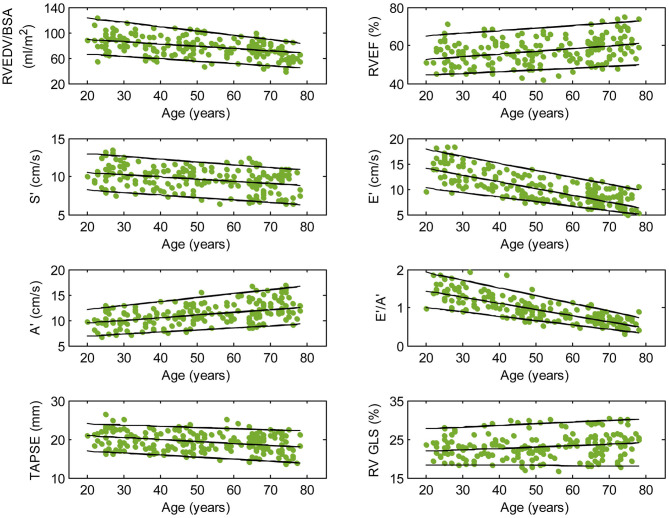
Right ventricular parameters plotted against age in male subjects. (Central line) The 50th percentile; (Top line) The 95th percentile; (Bottom line) The 5th percentile.

**Figure 3:**
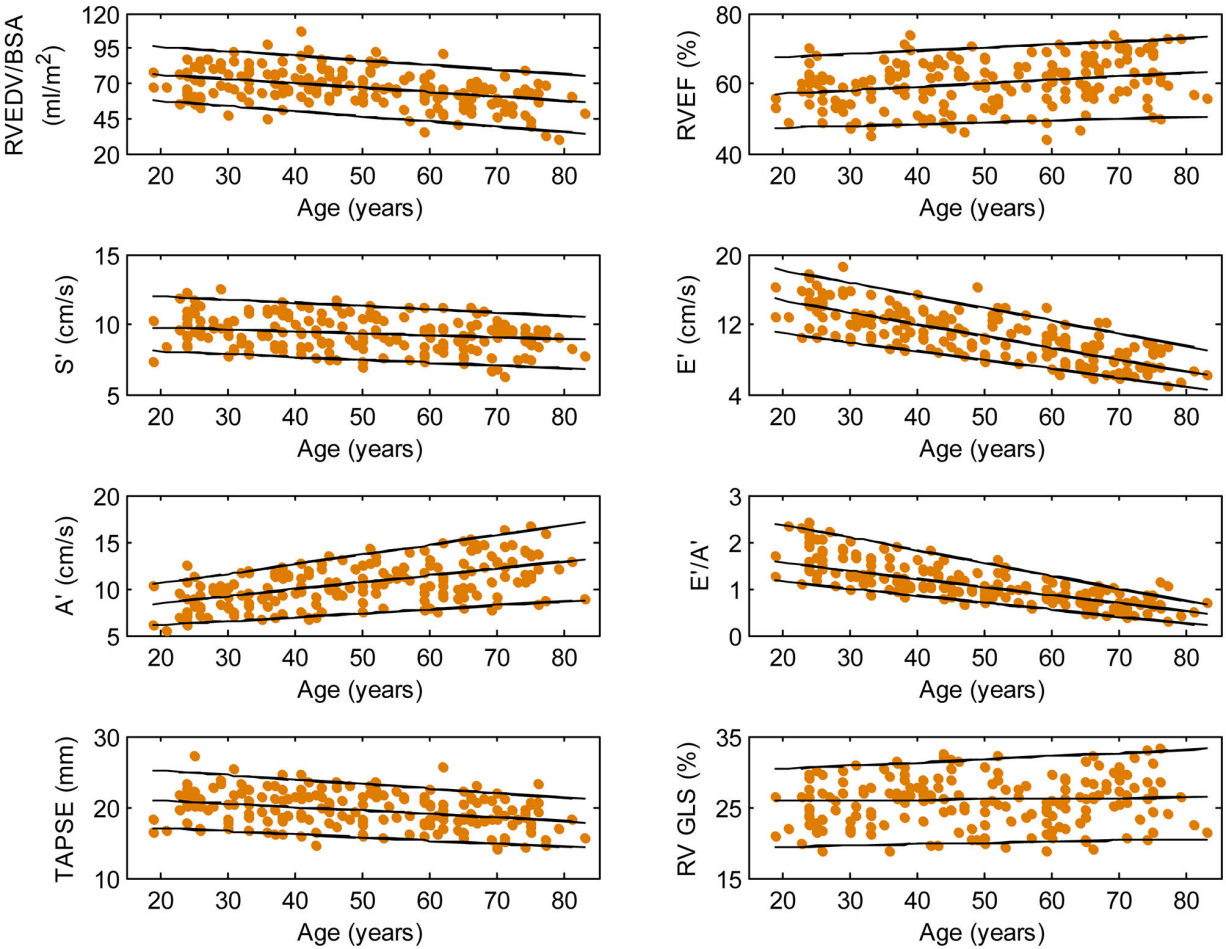
Right ventricular parameters plotted against age in female subjects. (Central line) The 50th percentile; (Top line) The 95th percentile; (Bottom line) The 5th percentile.

**Figure 4:**
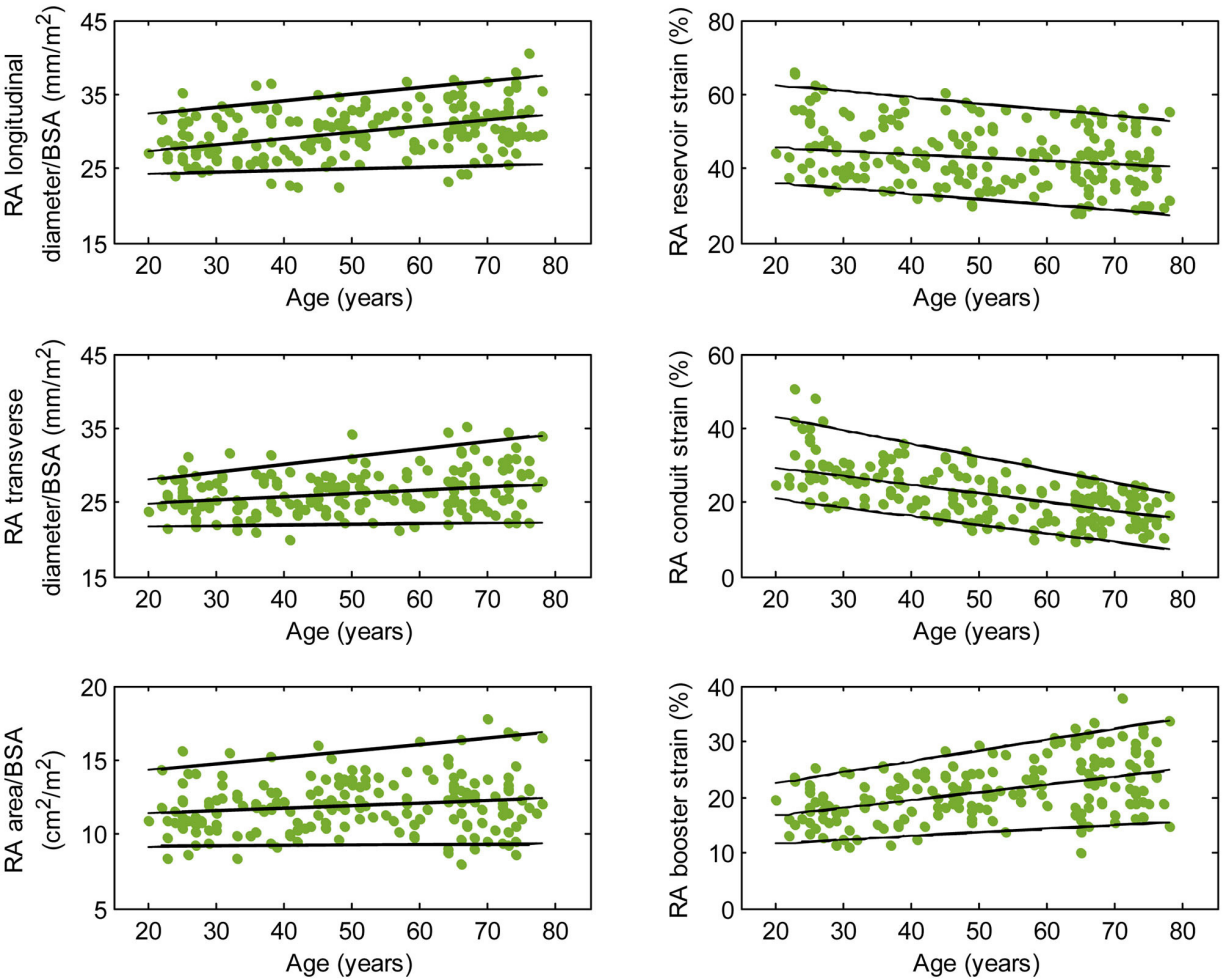
Right atrial parameters plotted against age in male subjects. (Central line) The 50th percentile; (Top line) The 95th percentile; (Bottom line) The 5th percentile.

**Figure 5:**
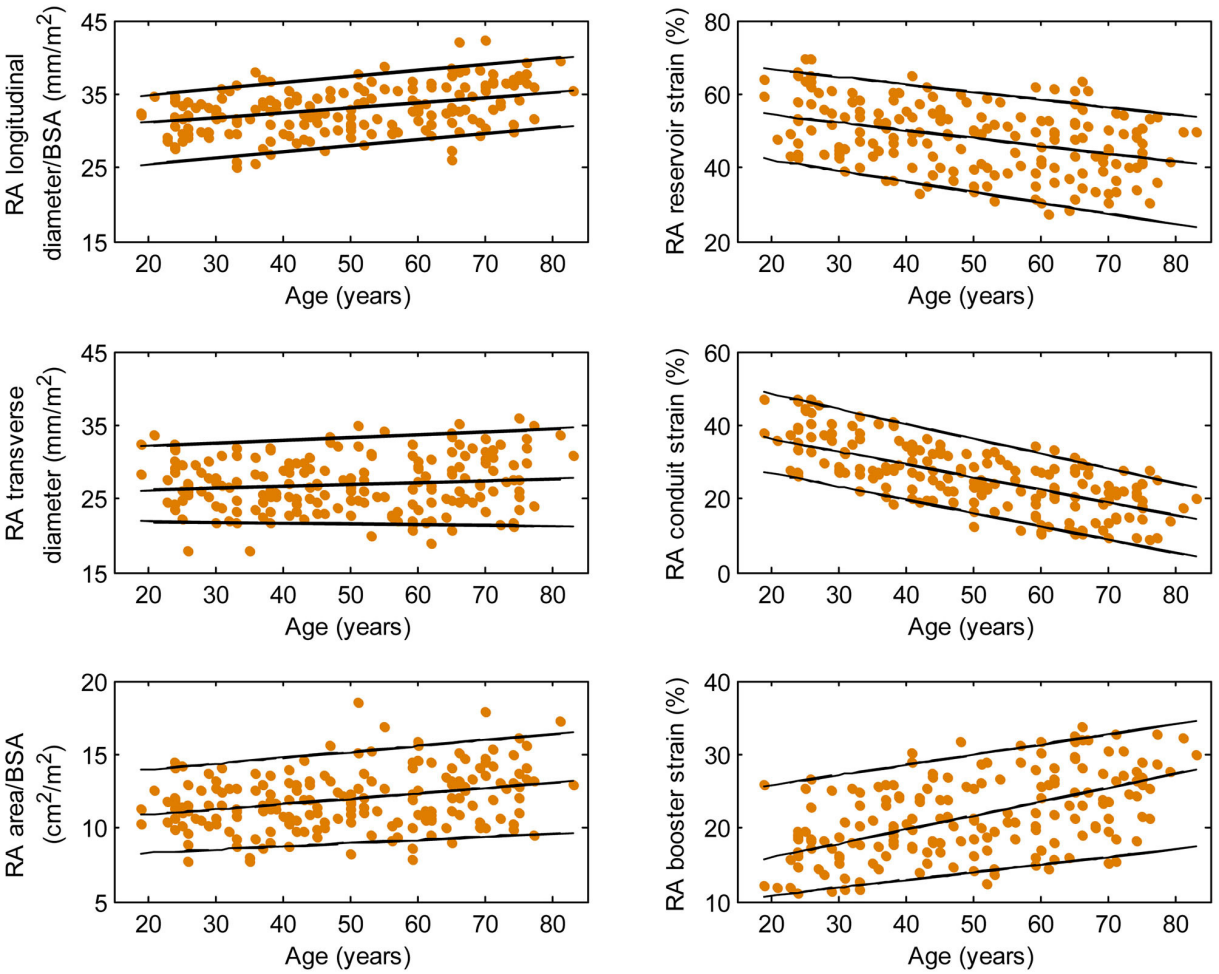
Right atrial parameters plotted against age in female subjects. (Central line) The 50th percentile; (Top line) The 95th percentile; (Bottom line) The 5th percentile.

We recruited 360 healthy individuals (male:female 180:180, age range 21–79 years). Baseline demographics and right heart CMR measurements stratified by sex are given in Table 1. Age group-specific CMR measurements stratified by sex are presented in Tables 2, 3. Figures 2, 3 show RV volume and systolic and diastolic RV function parameters plotted against age with 5th, 50th, and 95th percentile values in males and females, respectively. Figures 4, 5 show RA dimensions and phasic function parameters vs. age with 5th, 50th, and 95th percentile values in males and females, respectively. Measurements of right heart dimensions and volumes stratified by sex and age group are presented in Supplementary Tables 1–3.

**Table 1:** Demographics and right heart function parameters of the control population.

**Parameters**	**Total (*n* = 360)**	**Men (*n* = 180)**	**Women (*n* = 180)**	***P*-value**
**Demographics**				
Age (years)	50 ± 17	51 ± 17	49 ± 17	0.464
Height (cm)	163 ± 9	169 ± 7	158 ± 6	<0.0001
Weight (kg)	61 ± 11	66 ± 10	56 ± 9	<0.0001
BSA (m^2^)	1.7 ± 0.2	1.8 ± 0.2	1.6 ± 0.2	<0.0001
BMI (kg/m^2^)	22.8 ± 2.8	23.3 ± 2.7	22.4 ± 2.8	0.002
Systolic blood pressure (mmHg)	124 ± 12	127 ± 10	120 ± 12	<0.0001
Diastolic blood pressure (mmHg)	76 ± 9	78 ± 7	74 ± 10	<0.0001
Heart rate (beats per minute)	74 ± 11	74 ± 12	73 ± 11	0.881
**Right ventricular function (systolic and diastolic)**				
RVEF (%)	58 ± 7	57 ± 7	60 ± 7	0.001
S′ (cm/s)	9.5 ± 1.4	9.6 ± 1.6	9.4 ± 1.2	0.070
E′ (cm/s)	10.5 ± 2.9	10.3 ± 3.0	10.8 ± 2.9	0.138
A′ (cm/s)	10.9 ± 2.3	11.2 ± 2.2	10.5 ± 2.4	0.004
E′/A′	1.0 ± 0.4	1.0 ± 0.4	1.1 ± 0.5	0.001
TAPSE (mm)	19.5 ± 2.6	19.3 ± 2.6	19.7 ± 2.5	0.118
RV GLS (%)	24 ± 4	23 ± 3	26 ± 3	<0.0001
RV GLSR_s_ (1/s)	1.3 ± 0.3	1.3 ± 0.3	1.3 ± 0.2	0.052
RV GLSR_e_ (1/s)	1.5 ± 0.4	1.3 ± 0.4	1.6 ± 0.5	<0.0001
RV GLSR_a_ (1/s)	1.2 ± 0.4	1.2 ± 0.4	1.2 ± 0.4	0.703
**Right atrial phasic function**				
Reservoir strain ε_*s*_ (%)	46 ± 9	43 ± 8	48 ± 9	<0.0001
Conduit strain ε_*e*_ (%)	24 ± 8	22 ± 7	26 ± 9	<0.0001
Booster strain ε_*a*_ (%)	22 ± 5	21 ± 5	22 ± 6	0.408
Reservoir strain rate *SR*_s_ (1/s)	2.4 ± 0.5	2.3 ± 0.5	2.4 ± 0.5	0.019
Conduit strain rate *SR*_e_ (1/s)	−2.2 ± 0.8	−2.0 ± 0.7	−2.3 ± 0.8	0.001
Booster strain rate *SR*_a_ (1/s)	−2.7 ± 0.7	−2.7 ± 0.6	−2.8 ± 0.7	0.147

*Data are presented as mean ± SD. P-value is for t-test between sexes. BSA, body surface area; BMI, body mass index; RV, right ventricular; EF, ejection fraction; S′, peak systolic tricuspid annular velocity; E′, peak early diastolic tricuspid annular velocity; A′, peak late diastolic tricuspid annular velocity; TAPSE, tricuspid annular plane systolic excursion; GLS, global longitudinal strain; GLSR_s_, peak systolic global longitudinal strain rate; GLSR_e_, peak early diastolic global longitudinal strain rate; GLSR_a_, peak late diastolic global longitudinal strain rate*.

**Table 2:** Males: right heart function parameters by age group [mean ± SD (reference range, lower/upper limits calculated as mean ± 1.96·SD)].

**Parameters**	** <30 (*n* = 30)**	**30–39 (*n* = 30)**	**40–49 (*n* = 30)**	**50–59 (*n* = 30)**	**60–69 (*n* = 30)**	**≥70 (*n* = 30)**
**Right ventricular function (systolic and diastolic)**						
RVEF (%)	55 ± 7 (42, 68)	55 ± 7 (43, 68)	55 ± 6 (44, 66)	58 ± 7 (44, 71)	58 ± 7 (45, 71)	62 ± 7 (48, 76)
S′ (cm/s)	10.6 ± 1.7 (7.4, 13.9)	9.9 ± 1.2 (7.5, 12.4)	9.7 ± 1.4 (7.0, 12.4)	9.5 ± 1.5 (6.7, 12.4)	9.2 ± 1.6 (6.1, 12.3)	8.9 ± 1.5 (5.9, 11.9)
E′ (cm/s)	14.2 ± 2.6 (9.1, 19.2)	12.3 ± 1.9 (8.6, 16.0)	10.6 ± 2.1 (6.6, 14.7)	9.2 ± 1.6 (6.0, 12.3)	8.6 ± 1.7 (5.2, 11.9)	7.7 ± 1.7 (4.3, 11.0)
A′ (cm/s)	9.8 ± 1.7 (6.5, 13.0)	10.2 ± 1.6 (7.0, 13.4)	10.7 ± 1.8 (7.2, 14.3)	11.2 ± 1.7 (8.0, 14.5)	12.1 ± 2.3 (7.6, 16.6)	12.9 ± 1.9 (9.1, 16.8)
E′/A′	1.4 ± 0.3 (0.9, 1.9)	1.2 ± 0.2 (0.8, 1.7)	1.0 ± 0.3 (0.5, 1.5)	0.8 ± 0.2 (0.5, 1.1)	0.7 ± 0.2 (0.4, 1.0)	0.6 ± 0.1 (0.3, 0.9)
TAPSE (mm)	20.4 ± 2.8 (15.0, 25.8)	19.7 ± 2.3 (15.1, 24.2)	19.5 ± 2.5 (14.6, 24.4)	19.1 ± 2.4 (14.5, 23.8)	18.9 ± 2.4 (14.2, 23.6)	18.1 ± 2.8 (12.5, 23.6)
RV GLS (%)	23 ± 3 (18, 28)	23 ± 3 (17, 28)	22 ± 3 (16, 29)	23 ± 3 (17, 30)	23 ± 3 (17, 30)	24 ± 3 (17, 31)
RV GLSR_s_ (1/s)	1.3 ± 0.2 (0.9, 1.7)	1.2 ± 0.2 (0.8, 1.6)	1.3 ± 0.3 (0.7, 1.8)	1.3 ± 0.3 (0.7, 1.8)	1.2 ± 0.3 (0.7, 1.8)	1.3 ± 0.3 (0.7, 1.9)
RV GLSR_e_ (1/s)	1.7 ± 0.3 (1.1, 2.4)	1.6 ± 0.3 (1.1, 2.1)	1.3 ± 0.3 (0.8, 2.0)	1.2 ± 0.3 (0.7, 1.8)	1.1 ± 0.3 (0.4, 1.8)	1.1 ± 0.2 (0.6, 1.6)
RV GLSR_a_ (1/s)	0.9 ± 0.2 (0.5, 1.3)	1.1 ± 0.3 (0.5, 1.6)	1.2 ± 0.3 (0.5, 1.8)	1.2 ± 0.2 (0.7, 1.7)	1.4 ± 0.4 (0.7, 2.1)	1.6 ± 0.3 (1.0, 2.1)
**Right atrial phasic function**						
Reservoir strain ε_*s*_ (%)	48 ± 9 (30, 66)	45 ± 8 (30, 61)	43 ± 8 (27, 58)	41 ± 8 (26, 57)	42 ± 8 (27, 58)	41 ± 8 (26, 57)
Conduit strain ε_*e*_ (%)	30 ± 8 (14, 47)	27 ± 5 (18, 36)	22 ± 6 (10, 34)	20 ± 5 (9, 30)	18 ± 5 (8, 28)	18 ± 4 (10, 26)
Booster strain ε_*a*_ (%)	18 ± 3 (11, 24)	19 ± 4 (10, 27)	20 ± 4 (13, 28)	22 ± 4 (14, 30)	24 ± 6 (13, 36)	24 ± 6 (12, 36)
Reservoir strain rate *SR*_s_ (1/s)	2.5 ± 0.5 (1.5, 3.4)	2.4 ± 0.5 (1.5, 3.3)	2.2 ± 0.5 (1.3, 3.2)	2.2 ± 0.5 (1.2, 3.1)	2.2 ± 0.5 (1.1, 3.3)	2.3 ± 0.6 (1.2, 3.4)
Conduit strain rate *SR*_e_ (1/s)	−2.9 ± 0.7 (−4.2, −1.6)	−2.5 ± 0.5 (−3.4, −1.6)	−2.0 ± 0.5 (−3.1, −1.0)	−1.7 ± 0.4 (−2.6, −0.9)	−1.6 ± 0.4 (−2.4, −0.7)	−1.5 ± 0.4 (−2.3, −0.7)
Booster strain rate *SR*_a_ (1/s)	−2.4 ± 0.6 (−3.5, −1.3)	−2.3 ± 0.5 (−3.3, −1.3)	−2.5 ± 0.4 (−3.3, −1.7)	−2.6 ± 0.6 (−3.7, −1.5)	−2.9 ± 0.7 (−4.4, −1.5)	−3.1 ± 0.7 (−4.4, −1.8)

*RV, right ventricular; EF, ejection fraction; S′, peak systolic tricuspid annular velocity; E′, peak early diastolic tricuspid annular velocity; A′, peak late diastolic tricuspid annular velocity; TAPSE, tricuspid annular plane systolic excursion; GLS, global longitudinal strain; GLSR_s_, peak systolic global longitudinal strain rate; GLSR_e_, peak early diastolic global longitudinal strain rate; GLSR_a_, peak late diastolic global longitudinal strain rate*.

**Table 3:** Females: right heart function parameters by age group [mean ± SD (reference range, lower/upper limits calculated as mean ± 1.96·SD)].

**Parameters**	** <30 (*n* = 30)**	**30–39 (*n* = 30)**	**40–49 (*n* = 30)**	**50–59 (*n* = 30)**	**60–69 (*n* = 30)**	**≥70 (*n* = 30)**
**Right ventricular function (systolic and diastolic)**						
RVEF (%)	58 ± 5 (48, 68)	58 ± 8 (43, 73)	58 ± 7 (43, 72)	58 ± 6 (46, 70)	62 ± 6 (50, 74)	63 ± 7 (50, 77)
S′ (cm/s)	10.0 ± 1.2 (7.7, 12.4)	9.6 ± 1.2 (7.2, 11.9)	9.5 ± 1.3 (7.0, 12.0)	9.2 ± 1.3 (6.7, 11.7)	9.0 ± 1.1 (6.9, 11.2)	8.8 ± 1.0 (6.9, 10.8)
E′ (cm/s)	14.5 ± 2.2 (10.2, 18.7)	12.1 ± 1.7 (8.6, 15.5)	11.5 ± 1.9 (7.7, 15.3)	10.5 ± 2.1 (6.4, 14.7)	8.7 ± 1.9 (4.9, 12.5)	7.5 ± 1.5 (4.7, 10.4)
A′ (cm/s)	8.5 ± 1.7 (5.1, 11.9)	9.6 ± 1.8 (6.0, 13.1)	10.2 ± 1.9 (6.4, 14.0)	11.1 ± 2.0 (7.2, 14.9)	11.5 ± 2.4 (6.7, 16.2)	12.3 ± 2.3 (7.8, 16.8)
E′/A′	1.7 ± 0.4 (1.0, 2.5)	1.3 ± 0.3 (0.7, 1.9)	1.2 ± 0.3 (0.6, 1.7)	1.0 ± 0.2 (0.5, 1.4)	0.8 ± 0.2 (0.4, 1.2)	0.6 ± 0.2 (0.3, 1.0)
TAPSE (mm)	20.8 ± 2.4 (16.0, 25.6)	20.1 ± 2.5 (15.2, 25.0)	20.2 ± 2.4 (15.4, 25.0)	19.7 ± 2.5 (14.8, 24.5)	19.3 ± 2.3 (14.8, 23.7)	18.0 ± 2.5 (13.2, 22.8)
RV GLS (%)	25 ± 3 (19, 32)	26 ± 3 (19, 33)	26 ± 4 (18, 34)	25 ± 3 (18, 32)	26 ± 3 (19, 32)	27 ± 4 (20, 34)
RV GLSR_s_ (1/s)	1.3 ± 0.2 (0.9, 1.7)	1.3 ± 0.2 (0.9, 1.7)	1.3 ± 0.3 (0.8, 1.8)	1.3 ± 0.2 (0.9, 1.8)	1.3 ± 0.3 (0.8, 1.9)	1.4 ± 0.3 (0.7, 2.0)
RV GLSR_e_ (1/s)	2.1 ± 0.4 (1.3, 2.8)	1.7 ± 0.3 (1.1, 2.3)	1.6 ± 0.4 (0.9, 2.4)	1.5 ± 0.5 (0.6, 2.4)	1.3 ± 0.5 (0.4, 2.2)	1.2 ± 0.3 (0.6, 1.8)
RV GLSR_a_ (1/s)	0.9 ± 0.2 (0.5, 1.3)	1.1 ± 0.3 (0.5, 1.7)	1.2 ± 0.3 (0.6, 1.8)	1.3 ± 0.3 (0.8, 1.8)	1.3 ± 0.4 (0.6, 2.1)	1.7 ± 0.4 (0.9, 2.4)
**Right atrial phasic function**						
Reservoir strain ε_*s*_ (%)	56 ± 8 (40, 73)	49 ± 7 (36, 63)	49 ± 9 (33, 66)	44 ± 9 (26, 62)	45 ± 10 (25, 65)	44 ± 7 (29, 58)
Conduit strain ε_*e*_ (%)	39 ± 6 (26, 51)	30 ± 6 (17, 43)	28 ± 6 (17, 39)	23 ± 6 (11, 35)	20 ± 7 (6, 35)	18 ± 5 (8, 29)
Booster strain ε_*a*_ (%)	18 ± 4 (9, 26)	19 ± 4 (10, 26)	22 ± 5 (12, 31)	21 ± 5 (11, 31)	25 ± 5 (15, 36)	25 ± 5 (16, 35)
Reservoir strain rate *SR*_s_ (1/s)	2.8 ± 0.6 (1.5, 4.0)	2.4 ± 0.5 (1.5, 3.3)	2.4 ± 0.5 (1.5, 3.4)	2.2 ± 0.4 (1.5, 3.0)	2.4 ± 0.5 (1.3, 3.4)	2.4 ± 0.4 (1.6, 3.2)
Conduit strain rate *SR*_e_ (1/s)	−3.4 ± 0.7 (−4.7, −2.1)	−2.6 ± 0.5 (−3.7, −1.6)	−2.5 ± 0.5 (−3.5, −1.5)	−2.1 ± 0.5 (−3.2, −1.0)	−1.7 ± 0.5 (−2.7, −0.7)	−1.5 ± 0.4 (−2.3, −0.8)
Booster strain rate *SR*_a_ (1/s)	−2.5 ± 0.5 (−3.5, −1.4)	−2.5 ± 0.7 (−3.8, −1.2)	−2.8 ± 0.6 (−3.9, −1.6)	−2.8 ± 0.7 (−4.2, −1.3)	−3.0 ± 0.9 (−4.7, −1.2)	−3.1 ± 0.7 (−4.5, −1.8)

*RV, right ventricular; EF, ejection fraction; S′, peak systolic tricuspid annular velocity; E′, peak early diastolic tricuspid annular velocity; A′, peak late diastolic tricuspid annular velocity; TAPSE, tricuspid annular plane systolic excursion; GLS, global longitudinal strain; GLSR_s_, peak systolic global longitudinal strain rate; GLSR_e_, peak early diastolic global longitudinal strain rate; GLSR_a_, peak late diastolic global longitudinal strain rate*.

### Validation of Fast CMR Feature Tracking-Derived Measurements

Results from a sub-study of 60 subjects comprising 10 randomly selected from each of the six age groups showed that fast CMR feature tracking-derived TA dynamic measurements, RA and RV strain measurements were in good agreement with echocardiographic (*r* = 0.76–0.83, *P* < 0.0001, Supplementary Figure 1) and conventional CMR endocardial tracking-derived results (*r* = 0.75–0.94, *P* < 0.0001, Supplementary Figures 2–4).

### Influence of Sex and Age on Right Ventricular Function

Among RV systolic functional parameters, RVEF and RV GLS were significantly higher in females than in males but there were no significant sex differences in S′, TAPSE, and RV GLSR_s_ (Table 1). RVEF correlated positively with age in both males (*r* = 0.31, *P* < 0.0001) and females (*r* = 0.30, *P* < 0.0001). S′ and TAPSE exhibited negative correlations with age in both males (S′: *r* = −0.36, *P* < 0.0001; TAPSE: *r* = −0.28, *P* < 0.0001) and females (S′: *r* = −0.30, *P* < 0.0001; TAPSE: *r* = −0.31, *P* < 0.0001). RV GLS and RV GLSR_s_ were not associated with age in both sexes (Figures 2, 3).

With respect to RV diastolic function, females had significantly lower A′ and higher E′/A′ ratios and RV GLSR_e_ compared to males, but no significant sex differences in E′ and RV GLSR_a_ were observed (Table 1). Among male subjects, E′ (*r* = −0.75, *P* < 0.0001), E′/A′ ratio (*r* = −0.80, *P* < 0.0001) and RV GLSR_e_ (*r* = −0.58, *P* < 0.0001) decreased significantly with age, whereas A′ (*r* = 0.49, *P* < 0.0001) and RV GLSR_a_ (*r* = 0.57, *P* < 0.0001) increased with age. Similar positive associations of E′, E′/A′, and RV GLSR_e_, and negative associations of A′ and RV GLSR_a_ with age were observed among females (all *P* < 0.0001) (Figures 2, 3).

### Influence of Sex and Age on Right Atrial Phasic Function

Females had higher RA reservoir strain, conduit strain, reservoir strain rate, and conduit strain rate compared to males, while RA booster strain and strain rate were similar (Table 1). RA reservoir strain, conduit strain, and strain rate correlated negatively with age among both males (reservoir strain: *r* = −0.26, *P* < 0.0001; conduit strain: *r* = −0.61, *P* < 0.0001; conduit strain rate: *r* = −0.70, *P* < 0.0001) and females (reservoir strain: *r* = −0.38, *P* < 0.0001; conduit strain: *r* = −0.70, *P* < 0.0001; conduit strain rate: *r* = −0.75, *P* < 0.0001). Booster strain and strain rate exhibited positive correlations with age among both males (booster strain: *r* = 0.46, *P* < 0.0001; booster strain rate: *r* = 0.42, *P* < 0.0001) and females (booster strain: *r* = 0.51, *P* < 0.0001; booster strain rate: *r* = 0.34, *P* < 0.0001) (Figures 4, 5).

### Influence of Sex and Age on Right Ventricular Dimensions

Absolute and indexed RV EDV, ESV, and SV were higher in males compared to females (*P* < 0.0001 for all). Males had larger absolute RV basal and mid-cavity diameters than females (both *P* < 0.0001) but not when normalized to BSA (Supplementary Table 1). RV volumes correlated negatively with age among both males (EDV: *r* = −0.50, *P* < 0.0001; ESV: *r* = −0.49, *P* < 0.0001) and females (EDV: *r* = −0.39, *P* < 0.0001; ESV: *r* = −0.41, *P* < 0.0001), with similar correlations after normalization to BSA (males, EDV/BSA: *r* = −0.43, *P* < 0.0001; ESV/BSA: *r* = −0.44, *P* < 0.0001; females, EDV/BSA: *r* = −0.45, *P* < 0.0001; ESV/BSA: *r* = −0.44, *P* < 0.0001). Absolute and indexed RV basal and mid-cavity diameters were not significantly associated with age in both sexes.

### Influence of Sex and Age on Right Atrial Dimensions

Males had larger absolute RA diameters and areas compared to females. After normalization to BSA, indexed RA longitudinal and transverse diameters were significantly smaller in males compared to females, while the indexed RA area was not significantly different (Supplementary Table 1). Among male subjects, age was positively correlated with indexed RA longitudinal diameter (*r* = 0.32, *P* < 0.0001). Among female subjects, age was positively correlated with indexed RA longitudinal diameter (*r* = 0.37, *P* < 0.0001) and indexed RA area (*r* = 0.30, *P* < 0.0001).

### Reproducibility

Good to excellent intra- and inter-observer reproducibility was observed for RV and RA dimensions and volumes, TA velocities and displacement, RV longitudinal strain and strain rates, and RA phasic strains and strain rates (Table 4).

**Table 4:** Intra- and inter-observer reproducibility for right heart dimension and function parameters.

**Parameters**	**Intra-observer (*****n*** **=** **20)**		**Inter-observer (*****n*** **=** **20)**	
	**Bias (limits of agreement)**	**CV, %**	**Bias (limits of agreement)**	**CV, %**
**Right ventricular dimension**				
RV basal diameter (mm)	0.1 (−1,7, 1.9)	1.6	−2.1 (−4.4, 0.2)	4.4
RV mid-cavity diameter (mm)	1.3 (−0.1, 2.6)	2.7	1.3 (−0.6, 3.2)	3.0
RVEDV (ml)	0.9 (−11.4, 13.2)	3.5	1.4 (−11.6, 14.5)	3.8
RVESV (ml)	−0.3 (−10.8, 10.2)	7.6	2.6 (−8.0, 13.2)	8.9
RVSV (ml)	1.3 (−7.4, 10.0)	4.2	−1.1 (−10.8, 8.6)	4.5
**Right atrial dimension**				
RA longitudinal diameter (mm)	−0.8 (−2.5, 0.9)	1.5	0.7 (−1.1, 2.6)	1.6
RA transverse diameter (mm)	−0.1 (−4.8, 4.7)	3.6	2.7 (−0.2, 5.5)	4.8
RA area (cm^2^)	0.1 (−1.0, 1.1)	1.9	1.2 (−0.5, 2.9)	5.2
**Right ventricular function (systolic and diastolic)**				
RVEF (%)	0.6 (−6.7, 7.9)	4.2	−1.7 (−9.1, 5.7)	4.5
S′ (cm/s)	−0.1 (−0.9, 0.7)	3.0	−0.2 (−1.3, 1.0)	4.4
E′ (cm/s)	−0.5 (−1.4, 0.5)	4.7	−0.5 (−2.0, 1.0)	6.6
A′ (cm/s)	−0.5 (−2.5, 1.5)	6.6	0.1 (−1.9, 2.0)	5.8
E′/A′	0.1 (−0.2, 0.3)	6.9	−0.1 (−0.3, 0.2)	10.3
TAPSE (mm)	0.1 (−1.2, 1.3)	2.3	0.1 (−1.0, 1.2)	2.1
RV GLS (%)	−0.1 (−1.5, 1.3)	1.9	−0.1 (−2.1, 1.9)	2.7
RV GLSR_s_ (1/s)	−0.02 (−0.16, 0.11)	3.9	−0.02 (−0.15, 0.10)	3.6
RV GLSR_e_ (1/s)	−0.01 (−0.24, 0.21)	4.5	−0.04 (−0.34, 0.25)	6.1
RV GLSR_a_ (1/s)	−0.03 (−0.20, 0.15)	5.5	−0.03 (−0.29, 0.24)	7.9
**Right atrial phasic function**				
ε_s_ (%)	0.3 (−2.9, 3.5)	2.9	0.9 (−4.2, 5.9)	4.7
ε_e_ (%)	0.4 (−2.5, 3.4)	5.5	0.3 (−3.5, 4.1)	6.8
ε_a_ (%)	−0.1 (−2.5, 2.4)	4.4	0.3 (−2.5, 3.2)	5.3
*SR*_s_ (1/s)	0.01 (−0.24, 0.26)	4.0	0.03 (−0.28, 0.33)	5.3
*SR*_e_ (1/s)	0.01 (−0.26, 0.27)	5.3	0.02 (−0.28, 0.32)	6.0
*SR*_a_ (1/s)	0.06 (−0.27, 0.39)	4.6	−0.03 (-0.38, 0.32)	4.8

*CV, coefficient of variation; RV, right ventricular; EDV, end-diastolic volume; ESV, end-systolic volume; SV, stroke volume; EF, ejection fraction; RA, right atrial; S′, peak systolic tricuspid annular velocity; E′, peak early diastolic tricuspid annular velocity; A′, peak late diastolic tricuspid annular velocity; TAPSE, tricuspid annular plane systolic excursion; GLS, global longitudinal strain; GLSR_s_, peak systolic global longitudinal strain rate; GLSR_e_, peak early diastolic global longitudinal strain rate; GLSR_a_, peak late diastolic global longitudinal strain rate; ε_s_, right atrial reservoir strain; ε_e_, right atrial conduit strain; ε_a_, right atrial booster strain; SR_s_, right atrial reservoir strain rate; SR_e_, right atrial conduit strain rate; SR_a_, right atrial booster strain rate*.

## Discussion

In this study, we investigated age- and sex-specific changes in CMR-based right heart dimensions and functional measurements in a large, healthy Asian population. Increased age was associated with impairment of S′ and E′ velocities, TAPSE, and RV GLSR_e_ and increases in A′ velocity and RV GLSR_a_, in both sexes. RV GLS and RV GLSR_s_ were not affected by age in both sexes. As age increased, RA reservoir and conduit strains and strain rates decreased, while RA booster strain and strain rate increased.

CMR is recommended for many patients at the time of the transition from pediatric to adult congenital heart disease programmes, and this is the gold standard for RV volume, EF, flow quantification, and assessment of extracardiac anatomy. Additionally, CMR is recommended in the presence of clinical deterioration, non-diagnostic echo findings, and prior to surgical or transcatheter intervention (20). Data on age and sex-specific CMR reference ranges for right heart function in an Asian population are lacking at present. This information is crucial to CMR clinical practice in Asia. Moreover, our CMR-based method is simple, reproducible, and easily implemented for efficient RA and RV function assessment.

This work is novel for a number of reasons. First, it comprises the largest Asian CMR cohort reported to date, enrolling participants from multiple centers to provide broad generalizability of results and high precision in sample estimates of mean and SD. Second, CMR parameters were stratified not only by sex but also by age categories across a broad age range of 21–79 years. The increased granularity of results is key to clinical implementation and interpretation in light of the sex- and age-related differences observed in many of the studied parameters. Third, this study is the first to report age- and sex-specific reference ranges for various recently published fast CMR feature tracking parameters used to assess RV systolic and diastolic function as well as phasic RA function.

### RV Functional Parameters

Asian subjects in our study have numerically smaller right heart size compared to Caucasians in another study (21) (RVEDV: 121 ± 34 vs. 154 ± 40 ml; indexed RVEDV: 72 ± 15 vs. 85 ± 17 ml/m^2^), which underscores the need for ethnic-specific reference values. Our study showed a negative correlation between RV volume and age and a positive correlation between RVEF and age, corroborating similar observations in Caucasian subjects (21). Beyond conventional RV volumes and EF, we quantitated TA dynamics and RV longitudinal strain and strain rates—measurements for assessing RV function in both systole and diastole—and reported their age- and sex-specific reference ranges. Increasing age was associated with reductions in TAPSE, S′, E′, E′/A′, and RV GLSR_e_ and increases in A′ and RV GLSR_a_ in both sexes. These results agree with prior studies involving echocardiographic TDI (22) and conventional feature tracking CMR with endocardial contour tracing (23). Of note, we found that RV GLS and RV GLSR_s_ were not associated with age, which suggests that they are suited to be applied clinically as markers of disease progression unaffected by chronological aging. Our study replicated prior findings of higher systolic functional performance in females than in males as determined by RVEF (21) and RV GLS (24).

### RA Functional Parameters

We previously demonstrated that RA function as assessed using our fast RA feature tracking method had important diagnostic and prognostic implications in patients with PAH (7). RA volume and function (i.e., emptying fraction) assessment using volumetric analysis requires additional cine CMR acquisitions of atrial short-axis images and post-acquisitional analysis, which are not routinely performed. The area-length method, through geometric assumptions, shortens analysis time slightly (calculation still has to be repeated at all time phases) but is dogged by accuracy and reproducibility issues (25). In our study, we characterized the RA reservoir, conduit, and booster pump functions using strain and strain rate parameters that can be reproducibly and expeditiously measured. These parameters have been shown to be useful for detecting RA dysfunction, RV decompensation, and monitoring disease progression in patients with PAH (7). Among the healthy subjects in our study, females had higher reservoir and conduit strains but similar booster strain compared to males. This is concordant with a previous study of Caucasian subjects (25). In addition, we found age-related changes in RA function manifested as a significant decrease in conduit strain and an increase in booster strain. We surmise the latter is necessary in order to maintain reservoir strain, which increased slightly with age. These results are in agreement with previous reports using speckle tracking echocardiography (26) and feature tracking CMR with endocardial contour tracing (27).

### Advantages of Fast CMR Feature Tracking Parameters

This is the first study to establish age- and sex-specific reference ranges for CMR-derived TA dynamic parameters, which are a prerequisite for clinical applicability and adoption. The CMR-derived TA velocities correlated significantly with TDI-derived measurements, with no angle dependence and excellent reproducibility. This corroborates our previous work which demonstrated good correlation and agreement between CMR-derived mitral annular velocities and TDI measurements (18).

In addition to TA dynamics, we presented simplified strain indices relating time-varying distances between TA medial and lateral points and a fixed anatomical point at the RV apex or RA roof. Good correlation between the simplified longitudinal strains and those obtained from conventional endocardial contour-based feature tracking CMR was demonstrated, which is consistent with the results of our prior studies involving patients with rTOF, PAH, and age- and sex-matched controls (7, 8). In fact, the accuracy of conventional endocardial feature tracking-derived longitudinal strain is degraded in subjects with vigorous TA motion, as contour tracking of the RV free wall segment adjacent to the tricuspid valve in the long-axis view becomes adversely affected (28). Compared to conventional contour-based strain measurements, the fast feature tracking strain measurements presented in this study are less dependent on RV and RA geometry, and more closely approximate the motion and reflect the function of longitudinal fibers in the RV, which are the greatest contributors to RV contraction (29). Our prior studies have shown that the simplified approach is not only faster but also more reproducible compared to the conventional CMR feature tracking approach (7, 8, 15, 16).

### Limitations

There were some limitations in the present study. First, as CMR examinations were not performed repeatedly on the same subjects over time, the associations between age and CMR parameters are cross-sectional, not longitudinal. Nonetheless, the cross-sectional study design is commonly used in studies to establish reference ranges and make inferences regarding relationships in support of further research and clinical studies. We need a longitudinal study with repeated CMR scans on the same individual, which can help us investigate how age affects the heart structural and functional changes. Second, RV mass was not assessed in this study as RV mass is usually not quantified in a routine assessment because of the thin RV myocardium in healthy subjects.

## Conclusions

We investigated age- and sex-related CMR measurements for right heart dimensions and function in a large Asian cohort that are of significant clinical and research utility in Asia. Using CMR, knowledge of age-, sex-, and ethnicity-specific distributions of right heart measurements should aid in the correct interpretation of disease states.

## Data Availability Statement

The original contributions presented in the study are included in the article/Supplementary Materials, further inquiries can be directed to the corresponding authors.

## Ethics Statement

The studies involving human participants were reviewed and approved by SingHealth Centralized Institutional Review Board. The patients/participants provided their written informed consent to participate in this study.

## Author Contributions

R-ST, PC, LT, MF, CO, WR, AK, TT, JY, JT, YC, and LZ conceived the study design. SL, JG, CG, and XZ analyzed data. SL, R-ST, PC, LT, MF, CO, WR, AK, TT, JY, JT, YC, and LZ interpreted results. JA performed statistical analysis. SL drafted manuscript. JG, R-ST, PC, LT, MF, CG, XZ, CO, JA, WR, AK, TT, JY, JT, YC, and LZ edited and revised manuscript. All authors read and approved the final manuscript.

## Conflict of Interest

The authors declare that the research was conducted in the absence of any commercial or financial relationships that could be construed as a potential conflict of interest.

## Abbreviations

A′: peak tricuspid annular late diastolic velocity
BSA: body surface area
CMR: cardiovascular magnetic resonance
ε_*a*_: right atrial booster strain
ε_*e*_: right atrial conduit strain
ε_*s*_: right atrial reservoir strain
E′: peak tricuspid annular early diastolic velocity
EDV: end-diastolic volume
EF: ejection fraction
ESV: end-systolic volume
GLS: global longitudinal strain
GLSR_a_, global longitudinal strain rate during late diastole, GLSR_e_: global longitudinal strain rate during early diastole
GLSR_s_: global longitudinal strain rate during systole
LV: left ventricle
PAH: pulmonary arterial hypertension
RA: right atrium
rTOF: repaired tetralogy of Fallot
RV: right ventricle
S′: peak tricuspid annular systolic velocity
SD: standard deviation
*SR*_*a*_: right atrial booster strain rate
*SR*_*e*_: right atrial conduit strain rate
*SR*_*s*_: right atrial reservoir strain rate
SV: stroke volume
TA: tricuspid annular
TAPSE: tricuspid annular plane systolic excursion
TDI: tissue Doppler imaging.

## References

[1] Sanz J Sánchez-Quintana D Bossone E Bogaard HJ Naeije R. Anatomy, function, and dysfunction of the right ventricle: JACC state-of-the-art review. J Am Coll Cardiol. (2019) 73:1463–82. 10.1016/j.jacc.2018.12.076 30922478

[2] Geva T. Is MRI the preferred method for evaluating right ventricular size and function in patients with congenital heart disease? MRI is the preferred method for evaluating right ventricular size and function in patients with congenital heart disease. Circ Cardiovasc Imaging. (2014) 7:190–7. 10.1161/CIRCIMAGING.113.000553 24449548 PMC4006374

[3] Schulz-Menger J Bluemke DA Bremerich J Flamm SD Fogel MA Friedrich MG. Standardized image interpretation and post processing in cardiovascular magnetic resonance: Society for Cardiovascular Magnetic Resonance (SCMR) Board of Trustees Task Force on Standardized Post Processing. J Cardiovasc Magn Reson. (2013) 15:35. 10.1186/1532-429X-15-35 23634753 PMC3695769

[4] Leng S Jiang M Zhao XD Allen JC Kassab GS Ouyang RZ. Three-dimensional tricuspid annular motion analysis from cardiac magnetic resonance feature-tracking. Ann Biomed Eng. (2016) 44:3522–38. 10.1007/s10439-016-1695-2 27436293

[5] Ito S McElhinney DB Adams R Bhatla P Chung S Axel L. Preliminary assessment of tricuspid valve annular velocity parameters by cardiac magnetic resonance imaging in adults with a volume-overloaded right ventricle: comparison of unrepaired atrial septal defect and repaired tetralogy of Fallot. Pediatr Cardiol. (2015) 36:1294–300. 10.1007/s00246-015-1160-2 25835201

[6] Maffessanti F Gripari P Pontone G Andreini D Bertella E Mushtaq S. Three-dimensional dynamic assessment of tricuspid and mitral annuli using cardiovascular magnetic resonance. Eur Heart J Cardiovasc Imaging. (2013) 14:986–95. 10.1093/ehjci/jet004 23341146

[7] Leng S Dong Y Wu Y Zhao XD Ruan W Zhang GC. Impaired CMR-derived rapid semi-automated right atrial longitudinal strain is associated with decompensated hemodynamics in pulmonary arterial hypertension. Circ Cardiovasc Imaging. (2019) 12:e008582. 10.1161/CIRCIMAGING.118.008582 31088152

[8] Leng S Tan RS Guo JJ Chai P Zhang GC Teo L. Cardiovascular magnetic resonance-assessed fast global longitudinal strain parameters add diagnostic and prognostic insights in right ventricular volume and pressure loading disease conditions. J Cardiovasc Magn Reson. (2021) 23:38. 10.1186/s12968-021-00724-5 33789701 PMC8015087

[9] De Siqueira MEM Pozo E Fernandes VR Sengupta PP Modesto K Gupta SS. Characterization and clinical significance of right ventricular mechanics in pulmonary hypertension evaluated with cardiovascular magnetic resonance feature tracking. J Cardiovasc Magn Reson. (2016) 18:39. 10.1186/s12968-016-0258-x 27306901 PMC4910232

[10] Xie E Yu R Ambale-Venkatesh B Bakhshi H Heckbert SR Soliman EZ. Association of right atrial structure with incident atrial fibrillation: a longitudinal cohort cardiovascular magnetic resonance study from the Multi-Ethnic Study of Atherosclerosis (MESA). J Cardiovasc Magn Reson. (2020) 22:36. 10.1186/s12968-020-00631-1 32434529 PMC7240918

[11] Kawut SM Lima JA Barr RG Chahal H Jain A Tandri H. Sex and race differences in right ventricular structure and function: the multi-ethnic study of atherosclerosis-right ventricle study. Circulation. (2011) 123:2542–51. 10.1161/CIRCULATIONAHA.110.985515 21646505 PMC3111939

[12] Koh AS Gao F Leng S Kovalik JP Zhao XD Tan RS. Dissecting clinical and metabolomics associations of left atrial phasic function by cardiac magnetic resonance feature tracking. Sci Rep. (2018) 8:8138. 10.1038/s41598-018-26456-8 29802321 PMC5970174

[13] Valsangiacomo Buechel ER Mertens LL. Imaging the right heart: the use of integrated multimodality imaging. Eur Heart J. (2012) 33:949–60. 10.1093/eurheartj/ehr490 22408035

[14] Maceira AM Cosín-Sales J Roughton M Prasad SK Pennell DJ. Reference right atrial dimensions and volume estimation by steady state free precession cardiovascular magnetic resonance. J Cardiovasc Magn Reson. (2013) 15:29. 10.1186/1532-429X-15-29 23566426 PMC3627628

[15] Leng S Tan RS Zhao XD Allen JC Koh AS Zhong L. Fast long-axis strain: a simple, automatic approach for assessing left ventricular longitudinal function with cine cardiovascular magnetic resonance. Eur Radiol. (2020) 30:3672–83. 10.1007/s00330-020-06744-6 32107604

[16] Leng S Tan RS Zhao XD Allen JC Koh AS Zhong L. Validation of a rapid semi-automated method to assess left atrial longitudinal phasic strains on cine cardiovascular magnetic resonance imaging. J Cardiovasc Magn Reson. (2018) 20:71. 10.1186/s12968-018-0496-1 30396356 PMC6219067

[17] Leng S Ge H He J Kong LC Yang YN Yan FH. Long-term prognostic value of cardiac MRI left atrial strain in ST-segment elevation myocardial infarction. Radiology. (2020) 296:299–309. 10.1148/radiol.2020200176 32544032

[18] Leng S Zhao XD Huang FQ Wong JI Su BY Allen JC. Automated quantitative assessment of cardiovascular magnetic resonance-derived atrioventricular junction velocities. Am J Physiol Heart Circ Physiol. (2015) 309:H1923–35. 10.1152/ajpheart.00284.2015 26408537

[19] Ouyang RZ Leng S Sun AM Wang Q Hu LW Zhao XD. Detection of persistent systolic and diastolic abnormalities in asymptomatic pediatric repaired tetralogy of Fallot patients with preserved ejection fraction: a CMR feature tracking study. Eur Radiol. (2021). 10.1007/s00330-020-07643-6 33492469

[20] Di Salvo G Miller O Babu Narayan S Li W Budts W Valsangiacomo Buechel ER. Imaging the adult with congenital heart disease: a multimodality imaging approach-position paper from the EACVI. Eur Heart J Cardiovasc Imaging. (2018) 19:1077–98. 10.1093/ehjci/jey102 30084968

[21] Petersen SE Aung N Sanghvi MM Zemrak F Fung K Paiva JM. Reference ranges for cardiac structure and function using cardiovascular magnetic resonance (CMR) in Caucasians from the UK Biobank population cohort. J Cardiovasc Magn Reson. (2017) 19:18. 10.1186/s12968-017-0327-9 28178995 PMC5304550

[22] Dalen H Thorstensen A Vatten LJ Aase SA Stoylen A. Reference values and distribution of conventional echocardiographic Doppler measures and longitudinal tissue Doppler velocities in a population free from cardiovascular disease. Circ Cardiovasc Imaging. (2010) 3:614–22. 10.1161/CIRCIMAGING.109.926022 20581050

[23] Peng JP Zhao XD Zhao L Fan ZM Wang Z Chen H. Normal values of myocardial deformation assessed by cardiovascular magnetic resonance feature tracking in a healthy Chinese population: a multicenter study. Front Physiol. (2018) 9:1181. 10.3389/fphys.2018.01181 30233388 PMC6129778

[24] Qu YY Li H Rottbauer W Ma GS Buckert D Rasche V. Right ventricular free wall longitudinal strain and strain rate quantification with cardiovascular magnetic resonance based tissue tracking. Int J Cardiovasc Imaging. (2020) 36:1985–96. 10.1007/s10554-020-01895-5 32462446 PMC7497525

[25] Maceira AM Cosin-Sales J Prasad SK Pennell DJ. Characterization of left and right atrial function in healthy volunteers by cardiovascular magnetic resonance. J Cardiovasc Magn Reson. (2016) 18:64. 10.1186/s12968-016-0284-8 27719670 PMC5056480

[26] Peluso D Badano LP Muraru D Dal Bianco L Cucchini U Kocabay G. Right atrial size and function assessed with three-dimensional and speckle-tracking echocardiography in 200 healthy volunteers. Eur Heart J Cardiovasc Imaging. (2013) 14:1106–14. 10.1093/ehjci/jet024 23423966

[27] Truong VT Palmer C Young M Wolking S Ngo TNM Sheets B. Right atrial deformation using cardiovascular magnetic resonance myocardial feature tracking compared with two-dimensional speckle tracking echocardiography in healthy volunteers. Sci Rep. (2020) 10:5237. 10.1038/s41598-020-62105-9 32251322 PMC7089993

[28] Bhave NM Visovatti SH Kulick B Kolias TJ McLaughlin VV. Right atrial strain is predictive of clinical outcomes and invasive hemodynamic data in group 1 pulmonary arterial hypertension. Int J Cardiovasc Imaging. (2017) 33:847–55. 10.1007/s10554-017-1081-7 28168563 PMC5645792

[29] Brown SB Raina A Katz D Szerlip M Wiegers SE Forfia PR. Longitudinal shortening accounts for the majority of right ventricular contraction and improves after pulmonary vasodilator therapy in normal subjects and patients with pulmonary arterial hypertension. Chest. (2011) 140:27–33. 10.1378/chest.10-1136 21106653

