# Supplementary material for: Age- and Sex-Specific Changes in CMR Feature Tracking-Based Right Atrial and Ventricular Functional Parameters in Healthy Asians
Source: Front Cardiovasc Med. 2021 Jun 4;8:664431. doi: 10.3389/fcvm.2021.664431 (PMC8213369; doi:10.3389/fcvm.2021.664431)
Supplement: Supplementary file 1 [file Data_Sheet_1.PDF]

frontiers

Supplementary Material

Supplementary Table 1. Right heart dimension (absolute and normalized to BSA) parameters of the control population.

**Parameters**	**Total (n=360)**	**Men (n=180)**	**Women (n=180)**	***P* value**
**Right ventricular dimension**				
RV basal diameter (mm)	38±5	40±5	36±5	<0.0001
RV mid-cavity diameter (mm)	38±6	40±6	35±5	<0.0001
RVEDV (ml)	121±34	139±34	104±23	<0.0001
RVESV (ml)	52±20	60±21	43±13	<0.0001
RVSV (ml)	70±17	78±17	62±13	<0.0001
RV basal diameter/BSA (mm/m^2)	23±3	23±3	24±4	0.005
RV mid-cavity diameter/BSA (mm/m^2)	23±3	23±3	23±3	0.909
RVEDV/BSA (ml/m^2)	72±15	78±15	67±13	<0.0001
RVESV/BSA (ml/m^2)	31±10	34±10	27±8	<0.0001
RVSV/BSA (ml/m^2)	42±8	44±8	39±7	<0.0001
**Right atrial dimension**				
RA longitudinal diameter (mm)	52±5	53±5	51±5	<0.0001
RA transverse diameter (mm)	44±6	46±6	42±4	<0.0001
RA area (cm^2)	20±4	21±4	19±3	<0.0001
RA longitudinal diameter/BSA (mm/m^2)	31±4	30±3	33±4	<0.0001
RA transverse diameter/BSA (mm/m^2)	27±4	26±3	27±4	0.031
RA area/BSA (cm^2/m^2)	12±2	12±2	12±2	0.986

Data are presented as mean±SD. *P* value is for *t*-test between sexes. RV: right ventricular; EDV: end-diastolic volume; ESV: end-systolic volume; SV: stroke volume; BSA: body surface area; RA, right atrial.

Supplementary Table 2. Males: Right heart dimension (absolute and normalized to BSA) parameters by age group (mean±SD (reference range, lower/upper limits calculated as mean±1.96 SD)).

**Parameters**	**<30 (n=30)**	**30–39 (n=30)**	**40–49 (n=30)**	**50–59 (n=30)**	**60–69 (n=30)**	**≥70 (n=30)**
**Right ventricular dimension**						
RV basal diameter (mm)	39±5 (30, 49)	40±5 (30, 50)	40±4 (31, 49)	40±4 (31, 49)	40±4 (32, 49)	38±4 (29, 46)
RV mid-cavity diameter (mm)	44±6 (33, 54)	42±5 (31, 52)	41±6 (29, 52)	41±6 (29, 53)	38±6 (26, 50)	36±5 (26, 46)
RVEDV (ml)	162±34 (94, 229)	148±32 (85, 211)	149±35 (81, 218)	138±28 (84, 193)	129±28 (75, 183)	108±22 (66, 150)
RVESV (ml)	74±24 (27, 121)	67±22 (25, 109)	68±21 (26, 110)	59±17 (26, 92)	55±16 (25, 86)	41±11 (19, 63)
RVSV (ml)	88±15 (58, 118)	81±14 (52, 109)	82±17 (48, 115)	79±17 (45, 113)	74±17 (40, 107)	67±15 (37, 97)
RV basal diameter/BSA (mm/m^2)	22±3 (17, 26)	22±3 (16, 28)	22±3 (17, 27)	23±3 (17, 28)	24±3 (19, 29)	23±3 (17, 29)
RV mid-cavity diameter/BSA (mm/m^2)	24±3 (18, 30)	23±3 (18, 28)	22±3 (17, 27)	23±3 (17, 29)	22±4 (15, 29)	22±3 (17, 28)
RVEDV/BSA (ml/m^2)	88±15 (58, 118)	82±16 (50, 114)	80±13 (54, 106)	78±15 (48, 108)	75±13 (49, 101)	66±12 (44, 89)
RVESV/BSA (ml/m^2)	40±12 (17, 63)	37±11 (15, 59)	36±9 (18, 55)	33±9 (15, 52)	32±8 (17, 48)	25±7 (12, 39)
RVSV/BSA (ml/m^2)	48±7 (34, 62)	45±8 (29, 60)	44±6 (31, 56)	44±10 (25, 64)	43±9 (25, 61)	41±8 (26, 56)
**Right atrial dimension**						
RA longitudinal diameter (mm)	52±5 (41, 62)	53±4 (45, 62)	53±6 (42, 64)	54±3 (48, 60)	52±7 (39, 65)	51±5 (41, 62)
RA transverse diameter (mm)	47±4 (39, 55)	47±5 (36, 57)	47±4 (39, 56)	47±5 (36, 57)	45±8 (30, 60)	45±6 (33, 56)
RA maximal area (cm^2)	21±3 (14, 28)	21±3 (16, 27)	22±4 (15, 29)	22±3 (17, 27)	20±4 (12, 29)	20±4 (13, 27)
RA longitudinal diameter/BSA (mm/m^2)	28±3 (23, 34)	30±4 (23, 37)	29±3 (23, 35)	31±3 (26, 36)	31±4 (24, 38)	32±4 (25, 39)
RA transverse diameter/BSA (mm/m^2)	26±2 (21, 30)	26±3 (20, 32)	26±2 (22, 30)	27±3 (21, 32)	26±4 (18, 35)	28±4 (19, 36)
RA maximal area/BSA (cm^2/m^2)	11±2 (8, 15)	12±2 (9, 15)	12±2 (9, 15)	12±1 (10, 15)	12±2 (8, 16)	12±2 (8, 17)

RV: right ventricular; EDV: end-diastolic volume; ESV: end-systolic volume; SV: stroke volume; BSA: body surface area; RA, right atrial.

Supplementary Table 3. Females: Right heart dimensional (absolute and normalized to BSA) and functional parameters by age group (mean±SD (reference range, lower/upper limits calculated as mean±1.96 SD)).

**Parameters**	**<30 (n=30)**	**30–39 (n=30)**	**40–49 (n=30)**	**50–59 (n=30)**	**60–69 (n=30)**	**≥70 (n=30)**
**Right ventricular dimension**						
RV basal diameter (mm)	35±4 (26, 43)	35±3 (28, 41)	36±4 (29, 43)	38±5 (27, 48)	37±5 (27, 47)	39±5 (29, 49)
RV mid-cavity diameter (mm)	36±5 (27, 45)	36±4 (28, 44)	35±5 (25, 46)	35±5 (26, 45)	34±5 (25, 44)	33±5 (23, 44)
RVEDV (ml)	108±22 (66, 151)	112±21 (72, 153)	116±21 (74, 157)	108±21 (67, 148)	96±19 (58, 135)	84±19 (47, 121)
RVESV (ml)	45±12 (22, 69)	47±13 (22, 72)	49±15 (21, 78)	45±12 (22, 68)	37±10 (16, 57)	31±10 (11, 51)
RVSV (ml)	63±12 (39, 87)	65±14 (37, 94)	67±12 (44, 89)	62±12 (39, 86)	60±12 (36, 83)	53±12 (30, 76)
RV basal diameter/BSA (mm/m^2)	23±2 (18, 28)	22±3 (16, 28)	23±2 (18, 28)	24±4 (15, 32)	24±4 (16, 32)	26±4 (17, 34)
RV mid-cavity diameter/BSA (mm/m^2)	24±3 (18, 30)	23±3 (17, 29)	23±3 (16, 29)	22±4 (15, 29)	22±3 (16, 28)	22±4 (14, 30)
RVEDV/BSA (ml/m^2)	71±11 (50, 92)	71±12 (47, 95)	74±12 (51, 97)	67±12 (43, 91)	61±10 (43, 80)	55±12 (32, 79)
RVESV/BSA (ml/m^2)	30±7 (16, 45)	30±9 (12, 48)	31±9 (13, 50)	28±7 (14, 43)	23±5 (13, 34)	21±7 (7, 34)
RVSV/BSA (ml/m^2)	41±6 (29, 53)	41±8 (26, 57)	42±6 (31, 54)	39±7 (25, 53)	38±7 (25, 51)	35±7 (21, 49)
**Right atrial dimension**						
RA longitudinal diameter (mm)	47±4 (40, 54)	50±4 (42, 58)	49±5 (40, 59)	51±6 (40, 63)	52±5 (43, 61)	54±5 (44, 64)
RA transverse diameter (mm)	41±4 (33, 50)	40±4 (32, 48)	42±4 (35, 50)	42±5 (33, 51)	42±5 (33, 52)	43±4 (34, 52)
RA maximal area (cm^2)	17±3 (12, 23)	18±2 (13, 22)	18±3 (13, 24)	19±3 (12, 26)	19±3 (13, 25)	20±3 (14, 27)
RA longitudinal diameter/BSA (mm/m^2)	31±2 (27, 36)	32±4 (25, 39)	31±3 (26, 37)	32±4 (24, 40)	33±3 (27, 40)	36±3 (30, 41)
RA transverse diameter/BSA (mm/m^2)	28±3 (21, 34)	26±4 (18, 33)	27±3 (21, 33)	26±4 (19, 34)	27±4 (20, 35)	29±4 (20, 37)
RA maximal area/BSA (cm^2/m^2)	11±2 (8, 14)	11±2 (8, 15)	12±2 (8, 15)	12±2 (7, 17)	12±2 (9, 16)	13±2 (9, 18)

RV: right ventricular; EDV: end-diastolic volume; ESV: end-systolic volume; SV: stroke volume; BSA: body surface area; RA, right atrial.

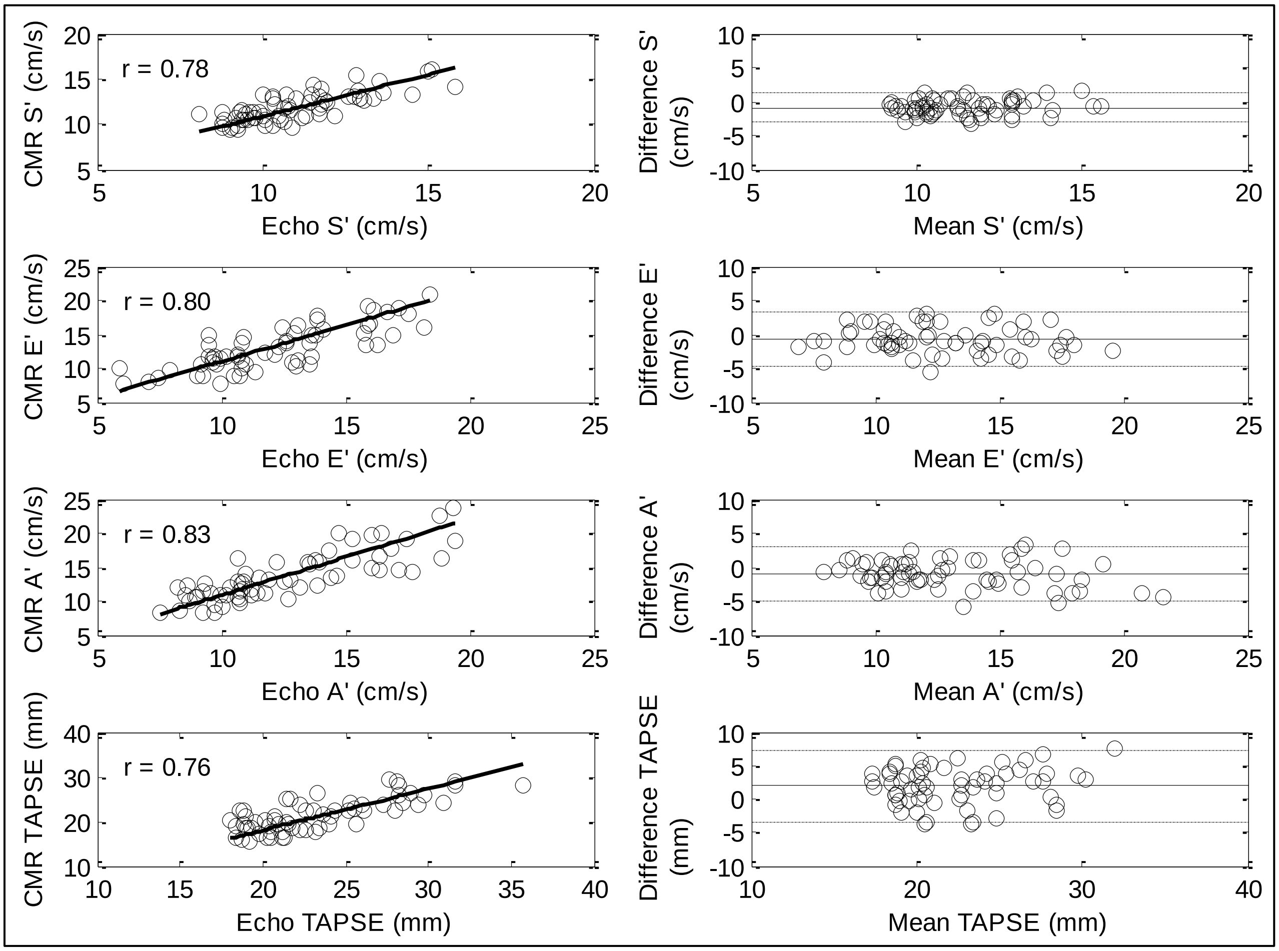

Supplementary Figure 1. Correlation and Bland-Altman plots for the **CMR** feature tracking-derived peak tricuspid annular velocities (systolic velocity S’, early diastolic velocity E’, late diastolic velocity A’) and tricuspid annular plane systolic excursion (TAPSE) compared with echocardiography **(Echo)**-derived results. In correlation plots, solid line denotes Passing-Bablok non-parametric regression line.

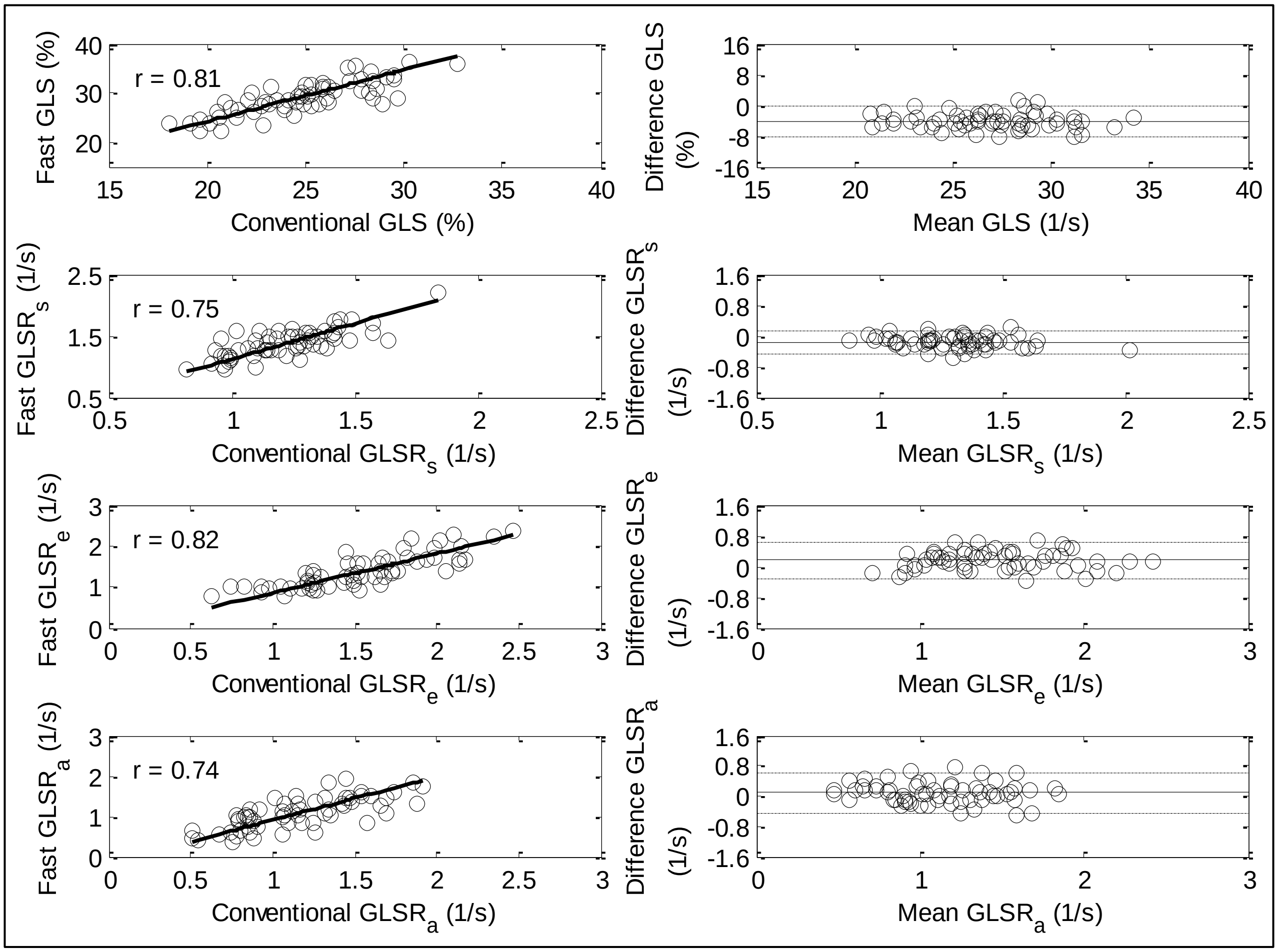

Supplementary Figure 2. Correlation and Bland-Altman plots for the **fast** feature tracking-derived right ventricular global longitudinal strain (GLS) and strain rates ($GLSR_s$, $GLSR_e$, $GLSR_a$) compared with **conventional** endocardial tracking-derived results. In correlation plots, solid line denotes Passing-Bablok non-parametric regression line.

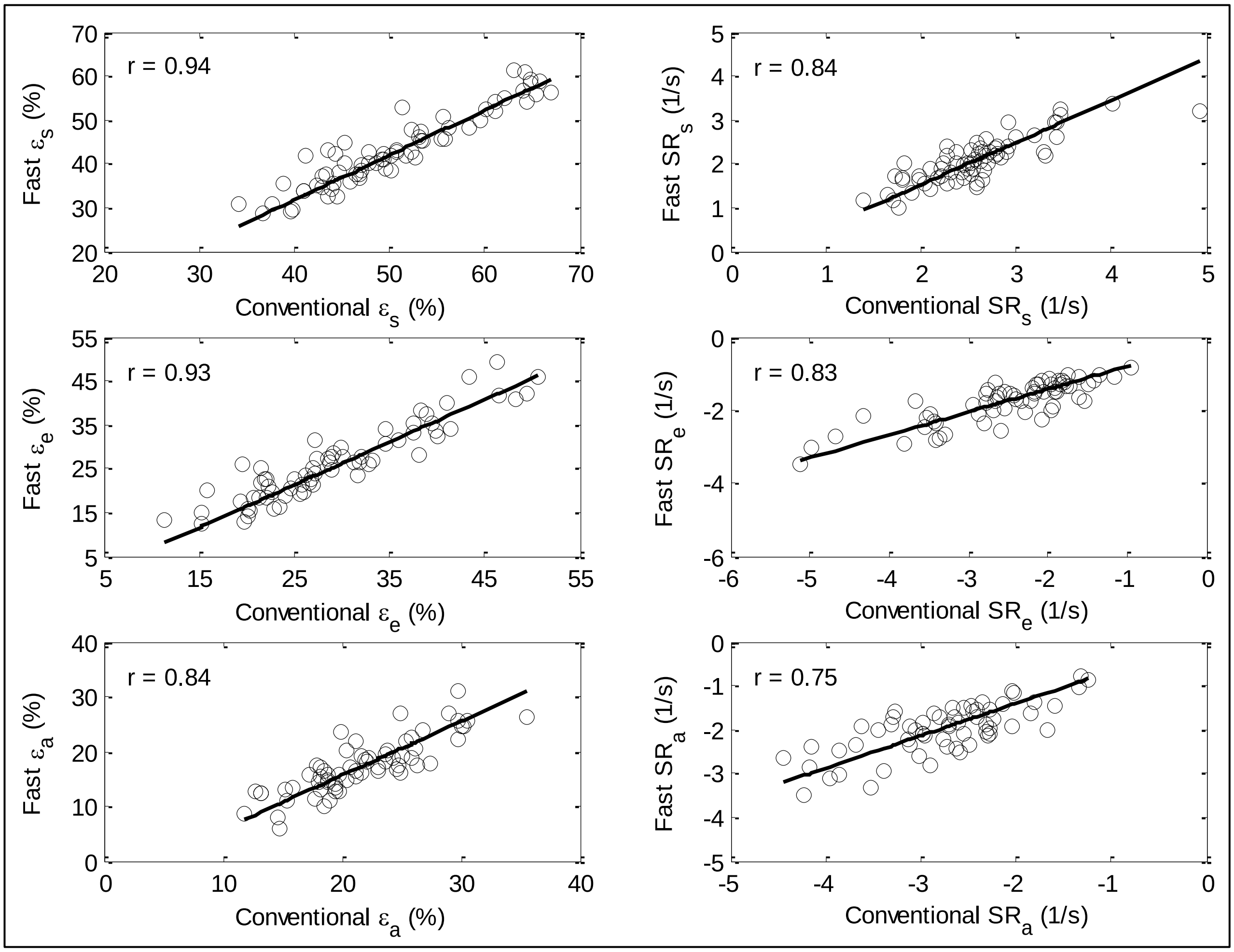

Supplementary Figure 3. Correlation plots for the **fast** feature tracking-derived right atrial phasic strains (reservoir strain ε_s, conduit strain ε_e, booster strain ε_a) and strain rates (reservoir strain rate SR_s, conduit strain rate SR_e, booster strain rate SR_a) compared with **conventional** endocardial tracking-derived results. Solid line denotes Passing-Bablok non-parametric regression line.

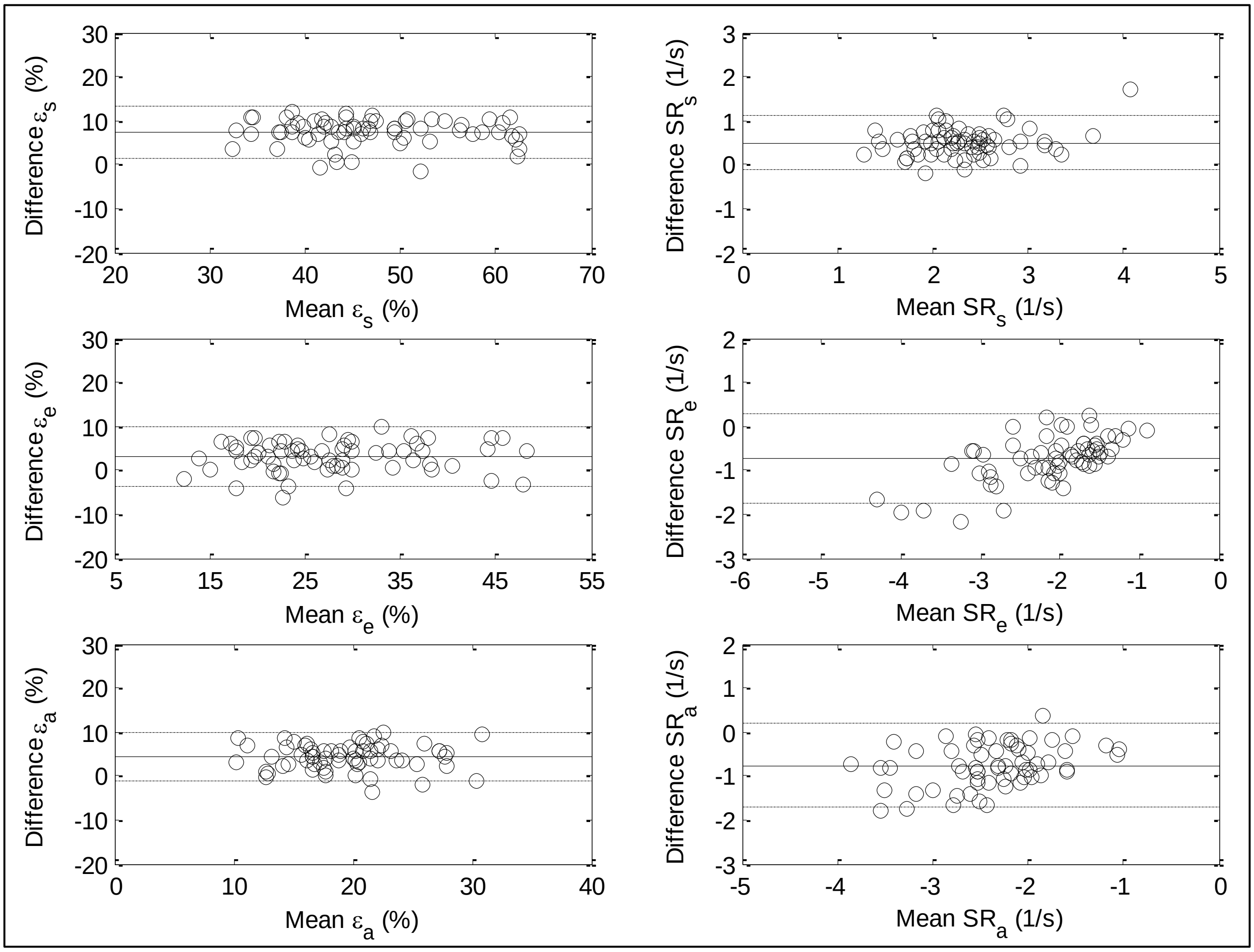

Supplementary Figure 4. Bland-Altman plots for the **fast** feature tracking-derived right atrial phasic strains (reservoir strain ε_s, conduit strain ε_e, booster strain ε_a) and strain rates (reservoir strain rate SR_s, conduit strain rate SR_e, booster strain rate SR_a) compared with **conventional** endocardial tracking-derived results.
