# Supplementary material for: Age- and Sex-Specific Changes in CMR Feature Tracking-Based Right Atrial and Ventricular Functional Parameters in Healthy Asians
Source: Front Cardiovasc Med. 2021 Jun 4;8:664431. doi: 10.3389/fcvm.2021.664431 (PMC8213369; doi:10.3389/fcvm.2021.664431)
Supplement: Supplementary file 2 [file Presentation_1.PPTX]

## Slide 1
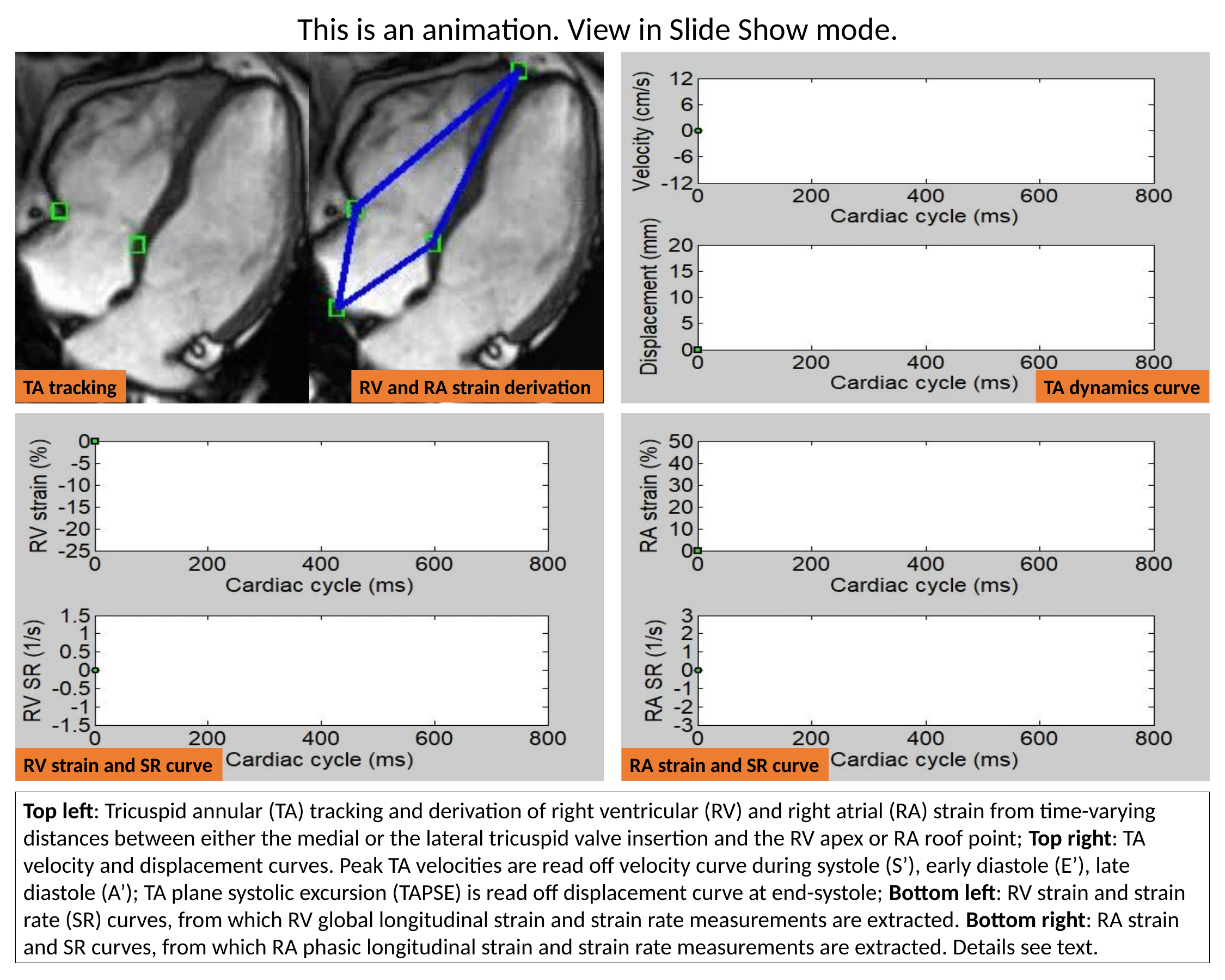

This is an animation. View in Slide Show mode.
TA tracking
RV and RA strain derivation
TA dynamics curve
RV strain and SR curve
RA strain and SR curve
Top left: Tricuspid annular (TA) tracking and derivation of right ventricular (RV) and right atrial (RA) strain from time-varying distances between either the medial or the lateral tricuspid valve insertion and the RV apex or RA roof point; Top right: TA velocity and displacement curves. Peak TA velocities are read off velocity curve during systole (S’), early diastole (E’), late diastole (A’); TA plane systolic excursion (TAPSE) is read off displacement curve at end-systole; Bottom left: RV strain and strain rate (SR) curves, from which RV global longitudinal strain and strain rate measurements are extracted. Bottom right: RA strain and SR curves, from which RA phasic longitudinal strain and strain rate measurements are extracted. Details see text.
